# How learning influences non-symbolic numerical processing: effects of feedback in the dot comparison task

**DOI:** 10.3389/fpsyg.2023.1287429

**Published:** 2024-01-30

**Authors:** Wiebke Hofmann, Annette Kinder, Judit Pekár

**Affiliations:** ^1^Institute of Psychology of Learning, Department of Education and Psychology, Free University of Berlin, Berlin, Germany; ^2^Department of Psychiatry and Neurosciences, Charité – Universitätsmedizin Berlin, Corporate Member of Freie Universität Berlin and Humboldt Universität Zu Berlin, Charité Campus Mitte (CCM), Berlin, Germany

**Keywords:** non-symbolic numerosity, learning, dot comparison, sensory integration, inhibition

## Abstract

It has long been debated how humans estimate the numerosity of sets of elements and what role continuous visual properties play in this process. The dot comparison task, in which the more numerous of two dot arrays must be selected, is a dominant method to investigate this phenomenon. It has been shown that the visual properties of the two dot patterns strongly influence the comparison. This influence can be systematically investigated by manipulating visual properties congruently and incongruently with numerosity. However, it remains unclear how learning and prior experience affect the influence of the visual properties. To address this question, we introduced feedback into the classical dot comparison task: during the learning phase, participants in the experimental group received feedback after each trial indicating whether their answer was correct whereas participants in the control group did not. After the learning phase, neither group received feedback. The convex hull of the dot patterns and the average dot diameter were manipulated congruently and incongruently with numerosity. Our results show that feedback had no effect on overall performance. However, when manipulated separately, dot diameter no longer affected performance in the experimental group after the learning phase, but it did in the control group. Moreover, this effect remained visible even when diameter and convex hull were manipulated simultaneously. This pattern of results is consistent with the notion of sensory integration which proposes that weights are assigned to different visual cues and that numerical judgments depend on an additive combination of these weights. We also found a correlation between performance on an arithmetic task and performance on trials in which dot size was manipulated incongruently with numerosity. However, there were no correlations between an inhibition task and performance in the dot comparison task. Taken together, the current results suggest that learning with feedback may affect some visual properties but not others. Future studies should further investigate a wider range of visual properties to examine which of them can be influenced by learning and under what conditions learning occurs.

## 1 Introduction

Numerical processing is essential in everyday life. Numerical comparisons and estimations are made many times a day and are needed to navigate simple situations such as choosing the queue with fewer people at the grocery store or selecting a bag that contains the desired number of fruits. Such situations require basic numerical processing that is thought to be supported by an innate number system, as even infants are able to process numerical stimuli when presented in non-symbolic notation (Brannon, 2002). It is widely believed that this innate ability serves as a foundation for the later acquired symbolic number system (Dehaene, 2001; Starr et al., 2013b).

As children develop, they become more proficient at processing non-symbolic numerosities. Not only their performance increases but they seem to rely less on non-numerical stimulus dimensions when estimating, discriminating and comparing the number of elements in visual dot displays or the number of tones in tone sequences (Halberda and Feigenson, 2008). However, the mechanisms responsible for the increase in proficiency during development are not yet fully understood. Not only brain maturation but also formal mathematical education seems to play a key role in the development of non-symbolic numerical abilities (Nys et al., 2013; Piazza et al., 2013). This suggests that the ability to estimate and compare non-symbolic numerosities is also influenced by learning processes.

Even in adulthood, learning processes may influence the way numerosities are processed. A study by Pekár and Kinder (2020), that examined non-symbolic numerical comparison, is relevant in this context. According to their study, the order of previous trials can influence the extent to which participants rely on non-numerical stimulus dimensions when making numerosity judgments. The influence of previous trials could be due to either transient attentional changes or a learning process. To date, however, there is no direct experimental evidence of learning in the numerical comparison task in adulthood. Therefore, the current study aims to investigate how learning alters the processing of numerical information in this task. To accomplish this, we added feedback to the dot comparison task in order to promote learning in adult participants and examined how the feedback affected the pattern of results, such as performance and the reliance on non-numerical stimulus dimensions.

### 1.1 Theoretical background

There are different theories that aim to describe the cognitive processes underlying numerical abilities. Early theories suggest the existence of an innate cognitive system that extracts numerosity from visual and other sensory scenes (Dehaene and Changeux, 1993; Verguts and Fias, 2004). This cognitive system, also known as ‘number sense’ or approximate number system (ANS), is believed to serve as the foundation for the later acquired symbolic number system and mathematical competency (Dehaene, 2007). The existence of such an evolutionary ancient number system is supported by findings demonstrating that even infants and animals can solve basic numerical comparisons and calculations (Agrillo et al., 2008; Izard et al., 2009; Rugani et al., 2011; Starr et al., 2013a). Studies using habituation paradigms and cross-modal matching paradigms with human infants support the innateness of this cognitive system by demonstrating the existence of abstract numerical representations from the very beginning of human life (Xu and Spelke, 2000; Izard et al., 2009; Coubart et al., 2014). Furthermore, strong evidence for the ANS comes from studies showing that the ability to discriminate numerosity obeys Weber’s law: it is more difficult to discriminate numerosities that are closer together than those that are further apart, and the minimal difference that can still be discriminated decreases with numerosity. This ability to select the more numerous of two dot arrays is referred to as the ANS acuity and can be formally quantified as the Weber fraction. Human infants as young as 6 months have been shown to have numerical abilities that obey Weber’s law (Starr et al., 2013a). Furthermore, it seems that ANS acuity not only increases during development, but also varies between individuals and is correlated with math achievement (Halberda et al., 2008; Starr et al., 2013b). This link, however, is still a matter of debate because findings regarding the relationship between non-symbolic numerosity processing and mathematical abilities have been inconclusive (Reynvoet et al., 2021). Whereas some studies found such a correlation (Halberda et al., 2008; Starr et al., 2013b), others failed to do so (Sasanguie et al., 2012, 2014). Reasons for these differences could be different measures of mathematical ability, different numerosity comparison tasks (Reynvoet et al., 2021), or possible moderating variables such as executive functions or inhibitory control (Cragg and Gilmore, 2014).

When investigating non-symbolic numerical processing, it is important to consider the visual properties which are interrelated with numerosity. These visual properties encompass features such as the physical size of the items, their distance to each other, and the area they cover. When all visual properties of two item arrays are the same, their numerosities are always equal. Consequently, when numerosity changes, the visual properties must also change (Gebuis and Reynvoet, 2012a). Therefore, the visual properties of a set of items contain important information about the numerosity and may serve as cues for making numerical comparisons and estimations (Gebuis et al., 2016; Gevers et al., 2016). For example, in order to choose the queue with fewer people at the grocery store, relevant visual properties could be the distance between the people or the total length of the queue, i.e., the shorter queue could be chosen by considering the length of the queue as well as how far apart people are standing. However, depending on the situation, visual properties may differ in their relevance for numerical processing. They may be helpful, uninformative, or even misleading for the estimation at hand. In the grocery store example, the total length of the queue may provide valuable information about the number of people because there is a natural correlation between numerosity and length in every day visual scenes like this one: Since more people take up more space, the longer queue typically has more people in it. However, it is important to note that visual properties of sets of elements cannot be viewed as isolated from each other. If the length of the queue is very large but so is the distance between the people, relying solely on the length of the queue can be misleading. In this case, the natural correlation between the length of the queue and numerosity is violated, i.e., the longer queue consists of less people. This example illustrates how visual cues can be either congruent or incongruent with numerosity. If the longer queue consists of more people, queue length is considered congruent with numerosity. However, if the people in one queue are standing very close to each other, making the more numerous queue shorter, then queue length is incongruent with numerosity. This example shows that whether an individual visual cue is helpful for numerosity comparison actually depends on the unique features of the item arrays.

Because of these natural correlations between numerosity and visual properties, researchers have devoted considerable effort into developing methods that can control for the relationship between numerosity and its confounding visual features (Dehaene et al., 2005; Gebuis and Reynvoet, 2011; Salti et al., 2016; De Marco and Cutini, 2020; Guillaume et al., 2020). As a result, while numerous studies support a pure number sense (Gallistel and Gelman, 2000; Feigenson et al., 2004; Verguts and Fias, 2004; Piazza et al., 2007; Cantlon et al., 2009; Stoianov and Zorzi, 2012), there is a growing body of evidence suggesting that the visual properties of item arrays are processed automatically and always influence numerical judgments to some extent even when they are irrelevant for the task (Gebuis and Reynvoet, 2012a,b,c; Leibovich et al., 2016; Pekár and Kinder, 2020). Therefore, an increasing number of studies have been devoted to investigate the exact nature of the relationship between continuous sensory cues and discrete numerosity. As a result, some researchers have come to question the notion of an innate number sense (Mix and Sandhofer, 2007), while others aimed to refine the theory of the ANS to accommodate such new findings (Nieder and Dehaene, 2009; Gevers et al., 2016; Leibovich et al., 2016).

The first evidence for the existence of such an influence of continuous visual features on numerosity comes from the conservation error. This is the phenomenon whereby young children under the age of seven usually interpret the longer line of objects as the more numerous one, whereas older children and adults do not make this error (Piaget, 1954). Although these findings have been refuted by subsequent research (Mehler and Bever, 1967) showing that children as young as 2 years old can choose the more numerous line regardless of line length, the main idea remains: visual properties do affect numerosity processing, and their influence seems to decrease over the course of development (Piazza et al., 2018). It is likely that this increase in the ability to estimate numerosity in visual displays is the result of a learning process.

When investigating the influence of visual properties on numerosity processing, usually dot comparison tasks are used (e.g., Gebuis and Reynvoet, 2012a; Leibovich and Henik, 2014; Pekár and Kinder, 2020). In these tasks, two dot arrays are presented to the participants and they are asked to judge which of the two arrays contains more dots. Usually, the visual properties of the dot arrays are manipulated to investigate their influence on numerosity processing. As mentioned before, visual properties can be either congruent or incongruent with numerosity. Accordingly, in the dot comparison task, a congruent manipulation means that the image with more dots also has larger visual cues, for example a larger convex hull (smallest contour around all dots), a larger average dot diameter, or a larger total surface area of dots. In contrast, incongruent manipulation means that the image with more dots has smaller visual properties, such as a smaller convex hull or smaller sized dots. In a set of dot array pairs, different visual properties can be manipulated independently of each other, so that, for example, one visual property is either congruent or incongruent with numerosity while the other visual property is held constant across dot arrays.

**Figure 1:**
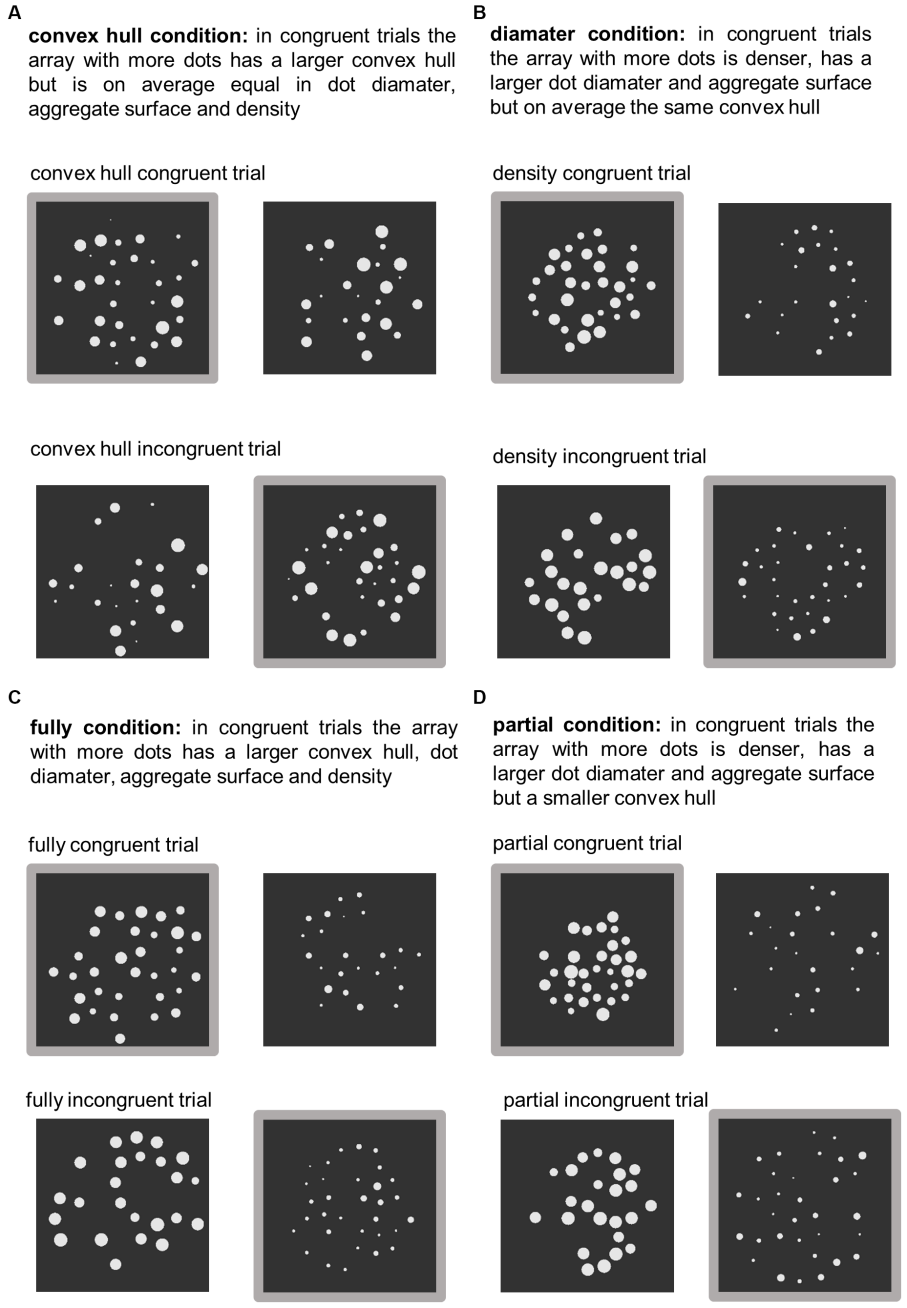
Examples of congruent and incongruent trials in the dot comparison task for each visual cue condition: **(A)** convex hull in/congruent, **(B)** diameter in/congruent, **(C)** fully in/congruent, and **(D)** partially in/congruent conditions. The figure shows examples of congruent and incongruent trials in the dot comparison task. For each of the four visual cue conditions, one congruent and one incongruent stimulus pair is shown. The more numerous stimulus is marked with gray border. Adapted from “The interplay between non-symbolic number and its continuous visual properties revisited: Effects of mixing trials of different types” by Pekár and Kinder (2020). Copyright 2019 by Experimental Psychology Society. Adapted with permission of the authors.

Gebuis and Reynvoet (2012a) used this method of independently manipulating visual properties to investigate how they affect numerosity processing. They manipulated the visual properties of the dot arrays in two different dimensions: their convex hull and their average dot diameter. Pairs of dot arrays were generated so that these visual cue dimensions were either congruent or incongruent with numerosity (see Figure 1 for example images). In two conditions, congruency of either convex hull or diameter was manipulated in isolation while the other visual cue was kept constant. This resulted in *convex hull congruent* and *convex hull incongruent* trial pairs (diameter was kept constant; *convex hull in/congruent* condition: Figure 1A) as well as *diameter congruent* and *diameter incongruent* trial pairs (convex hull was kept constant; *diameter in/congruent* condition: Figure 1B). In two further conditions, convex hull and diameter were manipulated simultaneously, either in the same or in opposite directions. Manipulating them in the same direction resulted in *fully congruent* (more numerous dot array has larger convex hull as well as larger dot diameter) and *fully incongruent* pairs of dot arrays (more numerous dot array has smaller convex hull and smaller average dot diameter; *fully in/congruent* condition: Figure 1C). Manipulating them in the opposite direction resulted in *partially congruent* and *partially incongruent trial* pairs (Figure 1D). It is important to note, that the terms *partially congruent* and *partially incongruent* are somewhat arbitrary because due to the opposite manipulation of the visual properties both kinds of dot array pairs were actually partially congruent and partially incongruent. In Gebuis and Reynvoet’s (2012a) study, the term *partially congruent* was assigned to dot array pairs which were *congruent* with respect to *diameter* and *incongruent* with respect to *convex hull*. Whereas the term *partially incongruent* was assigned to dot array pairs which were *incongruent* in *diameter* and *congruent* in *convex hull*. In this article we use the terms as introduced by Gebuis and Reynvoet (2012a). That is, in *partially congruent* trials, the more numerous dot array has a larger dot diameter but smaller convex hull, and in *partially incongruent* trials the more numerous dot array has a smaller dot diameter but larger convex hull. The same numerosities were used in each congruency and visual cue manipulation condition, and numerosity did not correlate with any of the visual properties of the dot array pairs either across all trials or within each visual cue manipulation method. Therefore, any observed congruency effects are the result of differences in how visual properties influence numerosity processing. The results showed that accuracy differed between congruent and incongruent trials in most visual cue conditions. Thus, although the visual cues were not informative of numerosity, they were still affecting the comparison to some extent. Interestingly, Gebuis and Reynvoet (2012a) found opposite congruency effects for convex hull and dot diameter. In the convex hull condition, accuracy was higher on congruent trials, i.e., when the more numerous dot array had a larger convex hull (diameter was kept constant). However, in the diameter condition accuracy was higher on incongruent trials, i.e., when the more numerous dot array had a *smaller* – rather than a larger – average dot diameter (convex hull was kept constant). Thus, manipulating convex hull congruently and incongruently with numerosity resulted in a *positive congruency effect* (larger convex hull is judged as more numerous, Table 1, row 1), while manipulating dot diameter congruently and incongruently with numerosity resulted in a *negative congruency effect* (smaller average dot size is judged as more numerous, Table 1, row 2). Even more interestingly, when these two properties were manipulated simultaneously, their opposite congruency effects were combined in an additive manner. When convex hull and diameter were manipulated together in the same direction in the *fully in/congruent* condition, their opposite effects on performance canceled each other out, as indicated by the absence of a congruency effect in this condition (Table 1, row 3). In the *partially in/congruent* condition, where diameter and convex hull were manipulated in opposite ways, an enhanced negative congruency effect was observed (Table 1, row 4). As in the *fully in/congruent* condition, this result can be explained in terms of an additive combination of the individual congruency effects, but the explanation is more complex. Note that in the *partially congruent* trials, only diameter was congruent, whereas convex hull was incongruent. Likewise, in the *partially incongruent* trials, only diameter was incongruent, while convex hull was congruent. The congruency effects in the *partially in/congruent* condition, i.e., the difference between partially congruent and partially incongruent trials, can be explained by combining the congruency effects of the convex hull and diameter conditions. To combine the congruency effects of convex hull and diameter, the positive congruency effect of convex hull must be reversed before being added to the negative congruency effect of diameter. Thus, the two effects combine to produce an enhanced negative effect in this condition.

**Table 1:** Congruency effects in the dot comparison task.

Visual cue condition	Congruency effect	Explanation
*Convex hull in/congruent*	Positive congruency effect **(+)**	Accuracy is higher on congruent than on incongruent trials.
*Diameter in/congruent*	Negative congruency effect **(−)**	Accuracy is higher on incongruent trials than on congruent trials.
*Fully in/congruent*	No congruency effect **(+)** + **(−)** → cancelation	Accuracy is equal on congruent and on incongruent trials. Convex hull and diameter are manipulated in the same direction, so their opposite effects cancel each other out when being added together.
*Partially in/congruent*	Increased congruency effect **(−)** + **(−)** → augmentation	Accuracy is around chance level on congruent trials but almost reaches a ceiling effect on incongruent trials. *Partially congruent* trials are congruent on diameter (−) and incongruent on convex hull (−). Thus, accuracy is especially low on these trials. In contrast, *partially incongruent* trials are incongruent on diameter (+) and congruent on convex hull (+). Thus, accuracy is very high on these trials. Through this opposite manipulation of convex hull and diameter, their opposing effects add up.

The table describes the congruency effects found in the different visual cue conditions of the dot comparison task by Gebuis and Reynvoet (2012a; see also Pekár and Kinder, 2020).

In summary, the pattern of congruency effects found by Gebuis and Reynvoet (2012a but see also Pekár and Kinder, 2020) shows that with this type of stimuli convex hull and dot diameter affect numerosity processing in opposite ways. Moreover, when convex hull and diameter are manipulated in either the same or opposite direction, the resulting congruency effects are a linear combination of the individual congruency effects. These results provide strong support for the idea that different visual cues can influence numerosity judgments simultaneously, and that their effects are combined through an additive process. This notion of integrating different visual properties during numerosity processing is referred to as Sensory Integration Theory (Gebuis et al., 2016; Gevers et al., 2016).

According to the Sensory Integration Theory, how much each visual property influences the judgment depends on the property’s weight. Weights differ in magnitude and can be positive (as in the case of convex hull) or negative (as in the case of diameter). In short, numerical judgments depend on the visual properties of the stimuli and on the weights of those properties. While the Sensory Integration Theory successfully explains how visual properties influence non-symbolic numerical processing, it does not explain how this process is initiated and developed. More specifically, it is unclear whether the weights given to the visual cues can be modified by learning. If the weights are indeed modified by learning, the values assigned to the weights should be influenced by the degree to which a particular visual cue is informative about numerosity. For example, if visual cues are less informative or even uninformative about numerosity, the weights assigned to the visual cues might decrease. Conversely, if visual cues are highly informative about numerosity, the weights might increase. A negative weight could result when the visual property and numerosity are negatively correlated: In this case, a strong expression of a visual property (such as *large* diameter) would be associated with *small* sets of items, whereas a weak expression of the property (such as *small* diameter) would be associated with *large* sets of items.

The Sensory Integration Theory makes no assumptions about the development of non-symbolic numerical processing and how learning might affect it. However, a comprehensive study by Piazza et al. (2018) provides a promising theoretical framework that could accommodate the notion of how learning might influence performance on the dot comparison task, possibly not only in childhood but also in adulthood. Piazza et al. (2018) compared performance and congruency effects in dot comparison tasks across different age groups and education levels. They suggest that two mechanisms could be responsible for the increase in accuracy in numerical comparison tasks that occur with development and education: *filtering* and *sharpening*. They argue that evidence for both hypotheses can be found in congruency effects. The *filtering* hypothesis suggests that the mechanisms for developmental improvements in numerical abilities lie in the increased ability to selectively focus on numerical information while ignoring related visual properties. Therefore, if the *filtering* hypothesis is correct, accuracy should mainly increase on incongruent trials, while accuracy on congruent trials should remain the same or even decrease, because participants rely less on covarying visual properties as their numerical ability increases. In contrast, the *sharpening* hypothesis suggests that the representation of number in the brain becomes more precise with maturation and education. Therefore, if the *sharpening* hypothesis is correct, overall accuracy should increase with maturation, i.e., accuracy should increase not only on incongruent but also on congruent trials. The results of Piazza et al. (2018) suggest that the improved ability in numerical comparison is the result of an improved ability to focus on number and ignore the non-numerical visual parameters, which is clearly in favor of the *filtering* hypothesis.

However, the authors do not provide a specific explanation as to which cognitive mechanism is responsible for the filtering process, e.g., whether it is domain-specific or domain-general. One possibility would be a domain-general inhibitory system that controls the interference of visual and numerical information (Houdé et al., 2011; Cappelletti et al., 2014). Another possibility is that *filtering* is domain-specific and, as a consequence, a domain-specific inhibitory mechanism is involved. It is also important to note that the study by Piazza et al. (2018) did not investigate learning directly, i.e., by experimental manipulation, such as introducing feedback in the numerical comparison task. Instead, the authors reanalyzed existing datasets from different age groups and educational levels and argued that the differences found between the groups were a result of learning. Thus, it remains an open question whether evidence for the *filtering* hypothesis can be found in an experimental setting that is directly designed to induce learning processes. Furthermore, it is unclear how the observed effects relate to different types of inhibition.

An important difference between the *filtering* account from Piazza et al. (2018) and the Sensory Integration Theory, is that in the former account, visual properties in non-symbolic numerosity processing are considered as a single dimension. Thus, this account does not consider the possibility that different visual properties may have different effects on non-symbolic numerosity processing. However, as discussed above, using the type of stimuli designed by Gebuis and Reynvoet (2012a) it has been shown that certain visual properties affect numerosity processing in opposite ways (Gebuis and Reynvoet, 2012a; Pekár and Kinder, 2020): When convex hull is manipulated, accuracy is higher on congruent trials, whereas when diameter is manipulated, accuracy is higher on incongruent trials. This is a robust finding and highlights the need to distinguish between individual visual properties as they appear to be processed in inherently different ways. Pekár and Kinder (2020) pointed out that the reverse congruency effect of dot diameter, in which smaller visual cues are judged to be more numerous, is difficult to explain at the neurobiological level. Therefore, the reverse congruency effect may be the result of a learning process, based on the perception of a negative correlation between the numerosity of the item set and the average item size.

Why is the positive congruency effect of convex hull in line with basic neural processes, while the negative congruency effect of diameter is not? Single-cell studies have revealed the existence of number-sensitive neurons in the monkey parietal cortex that are specialized to reacting to either the *more numerous* or the *less numerous* stimuli. Another population of neurons was found to respond to the visual dimension of the stimuli and responded to either the *larger* or the *smaller* stimuli. Interestingly, a third type of neuron responded to both the numerical and the visual dimensions of the stimuli. Some of these neurons responded to both more numerous stimuli and larger stimuli, while others responded to both less numerous stimuli and smaller stimuli. Thus, these neurons represented a positive correlation between magnitude and numerosity (Tudusciuc and Nieder, 2009; Eiselt and Nieder, 2013, 2014) and may explain positive congruency effects. However, there was no population of neurons that represented a negative correlation by responding to *less* on the visual dimension and at the same time to *more* on the numerical dimension. In summary, according to this pattern of results, the reverse congruency effect of diameter is difficult to explain at the basic neuronal level. However, this negative correlation can be found in many natural visual scenes. For example, a bag of a certain size filled with apples contains fewer pieces of fruit than a bag of the same size filled with blueberries. Similarly, a shopping cart filled with smaller items contains a greater number of items than a shopping cart filled with larger items. Repeated exposure to such scenes may induce a learning process that results in a set of items being perceived as more numerous when the average diameter of those items is smaller (Pekár and Kinder, 2020).

The notion that past experiences with the relationship between visual and numerical parameters affects numerical processing is supported by two studies that show trial history effects (Odic et al., 2014; Pekár and Kinder, 2020). For example, Pekár and Kinder’s (2020) study showed that trial history changes the magnitude of congruency effects in a dot comparison task. Different visual cue manipulation conditions – identical to those used by Gebuis and Reynvoet (2012a) – were presented to participants either in separate blocks or intermixed, i.e., trials of different visual cue manipulation methods were randomly altered. Congruency effects were significantly larger in the mixed condition than when presented in separate blocks. This suggests that the degree of reliance on visual cue can change depending on the immediately preceding experience. When only a single visual cue manipulation is used in the preceding trials, participants seem to learn (to some extent) that visual cues are not informative about numerosity. In the terminology of Sensory Integration Theory, the weights assigned to visual cues appear to be reduced in this condition. However, it is unclear whether learning processes are responsible for this effect or whether some transient factor, such as altered attention, has caused it. Therefore, the aim of the present study is to explicitly investigate learning processes in non-symbolic numerosity processing in young adults.

### 1.2 The present study

In the present study we aimed to investigate how learning affects non-symbolic numerosity comparison and the processing of visual properties related to numerosity. A simple way to promote learning is introducing feedback to a task where feedback is normally absent. Therefore, we modified the classical dot comparison task and introduced feedback by providing participants with objective information about numerosity. In particular, we were interested in determining how feedback affects the opposite congruency effects of convex hull and dot diameter when they are manipulated separately, as well as when they are manipulated simultaneously. Therefore, we used the visual cue manipulation method introduced by Gebuis and Reynvoet (2012a) and also used by Pekár and Kinder (2020). In addition, we were also interested in finding out whether and how this process is related to inhibitory skills and mathematical abilities.

To address all these goals, we divided the dot comparison task into three phases: *learning phase, test phase and transfer phase*. Participants were also assigned to one of two groups: the experimental group and the control group. Participants in the experimental group received feedback during the learning phase but not during the test phase and transfer phase. Participants in the control group received no feedback during the entire experiment. In the learning phase the visual cue conditions *convex hull in/congruent* and *diameter in/congruent* were used. In both conditions, only one visual property – either convex hull or diameter – is manipulated congruently or incongruently with numerosity within each trial, while the other visual property is kept constant (see the Methods section for more details). During the learning phase, the feedback given to participants of the experimental group after each trial indicated whether their response was correct or incorrect. As mentioned above, the control group did not receive any feedback. Since participants had to indicate which of two dot arrays was more numerous, the feedback reflected the numerosity of the trial pairs and was independent of the visual properties. As a result, participants had the opportunity to learn that visual properties do not contain information about numerosity throughout the experiment. Therefore, feedback in the learning phase may reduce or even eliminate the influence of one or both of the non-numerical properties on performance in the experimental group. During the test phase, participants were presented with the same visual cue conditions as during the learning phase, i.e., trials were *convex hull in/congruent* or *diameter in/congruent*. Neither group received feedback. By comparing performance and congruency effects between the two groups, we were able to assess whether the effect of the feedback persisted when feedback was omitted. Because within each trial either convex hull only or diameter only was manipulated, we could examine the effects of feedback separately for these visual cues. During the transfer phase, we wanted to assess whether the potential learning effects for convex hull and dot diameter are still present when these visual cues are manipulated simultaneously. If learning changes the weight assigned to individual visual properties, these changes should remain visible when the different properties are manipulated together. For this purpose, we used the *fully in/congruent* and *partially in/congruent* visual cue conditions. In both conditions convex hull as well as diameter were manipulated within each trial, either in the same or in opposite directions (see Methods section for more details). Thus, by comparing the performance of the control and feedback groups, we were able to assess how the separate learning effects for convex hull and diameter are integrated when both cues are manipulated together in the same and opposite directions. According to the Sensory Integration Theory the congruency effects, that have been altered through learning, should still combine in a linear fashion.

**Figure 2:**
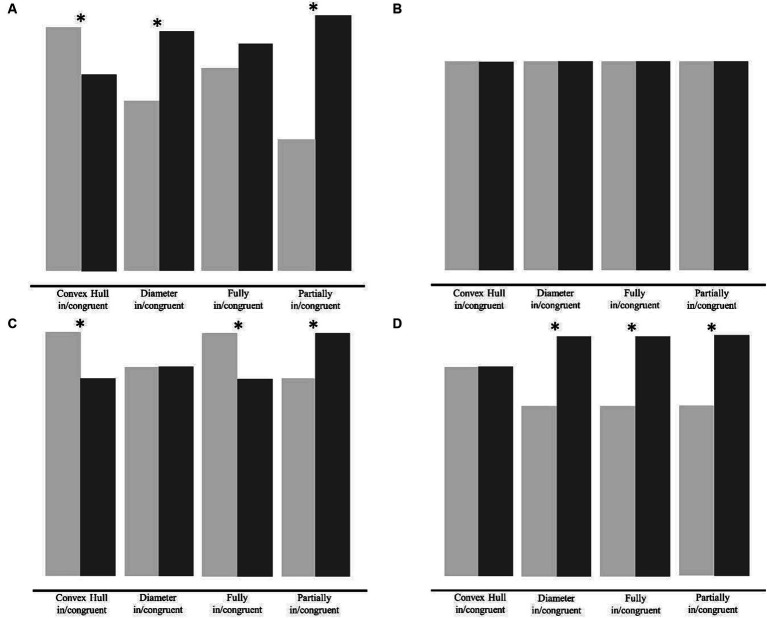
Schematic illustration of the possible outcomes in the experimental group. The figure shows the four possible outcomes we anticipated in the experimental group after the learning phase, i.e., the pattern of congruency effects in the test phase and transfer phase. **(A)** Pattern of congruency effects if the influence of diameter and convex hull are unchanged, **(B)** pattern of congruency effects if the influence of diameter as well as convex hull is eliminated, **(C)** pattern of congruency effects if the influence of diameter is selectively eliminated, and **(D)** pattern of congruency effects if the influence of convex hull effect is selectively eliminated. Asterisks denote congruency effects that are expected to be significant in the specific scenarios.

We anticipated four possible outcomes in the experimental group all of which are illustrated in Figure 2. First, if feedback in the learning phase has no effect on either convex hull or diameter, then the previously reported pattern of congruency effects will be found for both groups in both the test and transfer phases (Figure 2A; Gebuis and Reynvoet, 2012a; Pekár and Kinder, 2020). Second, if learning affects both visual cues, then feedback will reduce or eliminate the effects of both convex hull and diameter on numerical processing. If visual cues have no effect at all, no differences in accuracy between congruent and incongruent trials will be observed in any of the visual cue conditions in the feedback group (Figure 2B). Third, if learning affects only diameter, then the effect of diameter on numerosity comparison will be reduced or even eliminated. The convex hull effect, on the other hand, will still be present in all three visual cue conditions, in which convex hull is manipulated (*convex hull in/congruent, fully in/congruent* and *partially incongruent* conditions). Figure 2C shows the pattern of results that emerges when the diameter effect is selectively eliminated – there is no effect in the *diameter in/congruent* condition and an unchanged effect in the *convex hull in/congruent* condition. In the *fully in/congruent* condition, there is an enhanced effect, because the convex hull effect is no longer canceled out by the negative congruency effect of diameter. In the *partially in/congruent* condition there is a negative congruency effect because *partially congruent* trials are incongruent with respect to convex hull and *partially incongruent* trials are congruent with respect to convex hull. Fourth, if learning reduces or even eliminates the influence of convex hull only, then the congruency effect of convex hull will be reduced or eliminated (Figure 2D). In this case, a reverse congruency effect is expected in all visual cue conditions, in which diameter was manipulated (*diameter in/congruent, fully in/congruent, and partially incongruent* conditions).

To draw further conclusions about which underlying system is involved in the integration of various visual properties and numerical information, we looked at the role of inhibitory processes and mathematical abilities. Hence, we used a Color Stroop task (Bäumler, 1985) for measuring inhibitory skills and correlated the inhibition score with accuracies in the dot comparison task. We decided to use a Color Stroop task because we wanted to ensure that neither numerical nor magnitude aspects are measured in the inhibition task that could artificially inflate the correlation. Thus, we aimed at measuring inhibition related to domain-general rather than domain-specific processes. To investigate to what extent dot comparison abilities relate to math performances, we measured mathematical competence with a mental arithmetic task.

## 2 Methods

### 2.1 Participants

A total of 42 participants took part in the study. Two participants had to be excluded due to errors that occurred during data collection. This resulted in a sample size of 40 participants (11 male, 29 female, age 18–35 years, *M* = 23.65, *SD* = 4.12). Outliers were defined as performances that deviated more than two standard deviations from the group mean. No participant had to be excluded using this criterion.

All participants had normal or corrected-to-normal vision and normal color vision. Only healthy subjects (no neurological diseases, no major mental disorders, no diagnosed dyslexia, or dyscalculia) were allowed to participate in the study. Participants were mainly students including many psychology students. An email was distributed with information about the study and people could respond voluntarily to take part. All signed an informed consent form and were paid for their participation. Psychology students could alternatively receive course credit.

### 2.2 Apparatus and procedure

The study was conducted in the psychological laboratory of the Department of Education and Psychology at the Free University Berlin. The experiment consisted of three tasks: Color Stroop task, dot comparison task, and the arithmetic task. The Color Stroop task was paper-pencil based and the other two tasks were computer-based. The computer-based tasks were programmed using the PsychoPy software (Peirce et al., 2019). The experiment lasted approximately 90 minutes and participants had the opportunity to take breaks after each block and between tasks.

### 2.3 Color Stroop task

Inhibitory skills were measured using a paper-pencil version of the German Color Stroop task (Bäumler, 1985). The task consisted of three subtasks: (1) Reading color words, (2) Naming colored lines, and (3) Interference. First, participants had to read color words (green, blue, red, and yellow) printed in black as quickly as possible. Second, participants were presented with lines printed in different colors (green, blue, red, and yellow) and had to name the color of the lines as quickly as possible. The third subtask was a mixture of the first and second subtasks: Color words were printed in different colors and participants had to name the color of the print as quickly and accurately as possible while ignoring the meaning of the color word. Time was measured and mistakes were noted. Before completing the task, participants received a practice block. During the practice block they were presented with 15 trials of each subtask to ensure that they understood the instructions. After the practice blocks, they completed three parallel versions of each subtask while time was measured and errors were noted. This resulted in three measured times per subtask, the median of which was calculated. Completing the Stroop task took approximately 15 minutes.

Following the instructions in the manual, we computed a measure of inhibition called *selectiveness*, which represents the individual’s ability to ignore irrelevant stimuli (Bäumler, 1985). First, the median times of subtask 2 and subtask 3 were logarithmically transformed (log(x)100). Then, based on the logarithmic values from subtask 2, expectancy values for subtask 3 were taken from the general norm and subtracted from the observed logarithmic values of subtask 3. These differences were then transformed to *t*-values using the general norm and represented the individual’s inhibition score. This inhibition score was calculated for each participant and used in further analyses to provide a measure of inhibitory ability.

### 2.4 Dot comparison task

In the dot comparison task two sets of dot arrays were presented consecutively on a computer screen and participants were instructed to indicate with button press which image contained more dots. The stimuli used in this study were exactly the same as those used in the dot comparison study by Pekár and Kinder (2020) which were constructed in the same way as those used by Gebuis and Reynvoet (2012a). White dots were presented on a dark gray background. The dot size ranged from 0.11 to 0.79 degrees of visual angle.

Four visual properties were manipulated: convex hull (smallest contour around the dot array), average dot diameter, aggregate surface area of the dots, and density (aggregate surface divided by convex hull). However, the visual properties average dot diameter, aggregate surface, and density are highly correlated and cannot be manipulated independently of each other. For example, if the average dot diameter increases, the aggregate surface of dots and their density also increase, while convex hull may remain constant. Thus, average dot diameter and its related properties (aggregate surface of dots and density) were manipulated together in one visual cue condition while convex hull was manipulated independently of the other three properties. In the remainder of this article, we use the term diameter when referring to these three highly correlated properties. The four visual properties were either congruent or incongruent with numerosity. That is, on congruent trials, the manipulated visual property was greater in the dot array containing more dots, whereas on incongruent trials the manipulated visual property was greater in the dot array containing fewer dots (for further details on the stimuli see Pekár and Kinder, 2020).

This manipulation resulted in four different visual cue conditions (Figure 1): (a) *convex hull in/congruent*, (b) *diameter in/congruent*, (c) *fully in/congruent*, and (d) *partially in/congruent*. In the (a) *convex hull in/congruent* condition convex hull was either congruent or incongruent with numerosity while average dot diameter, aggregate surface area of the dots, and density were held constant. In the (b) *diameter in/congruent* condition average dot diameter, aggregate surface area, and density were either congruent or incongruent with numerosity while the convex hull was kept constant. In the (c) *fully in/congruent* condition, both convex hull and diameter (as well as its related properties) were manipulated in the same direction. Both were either congruent or incongruent with numerosity. In the (d) *partially in/congruent* condition, convex hull and diameter were manipulated in opposite directions. Specifically, *partially congruent* trials were congruent for diameter and incongruent for convex hull. *Partially incongruent* trials, however, were incongruent for diameter and congruent for convex hull.

**Figure 3:**
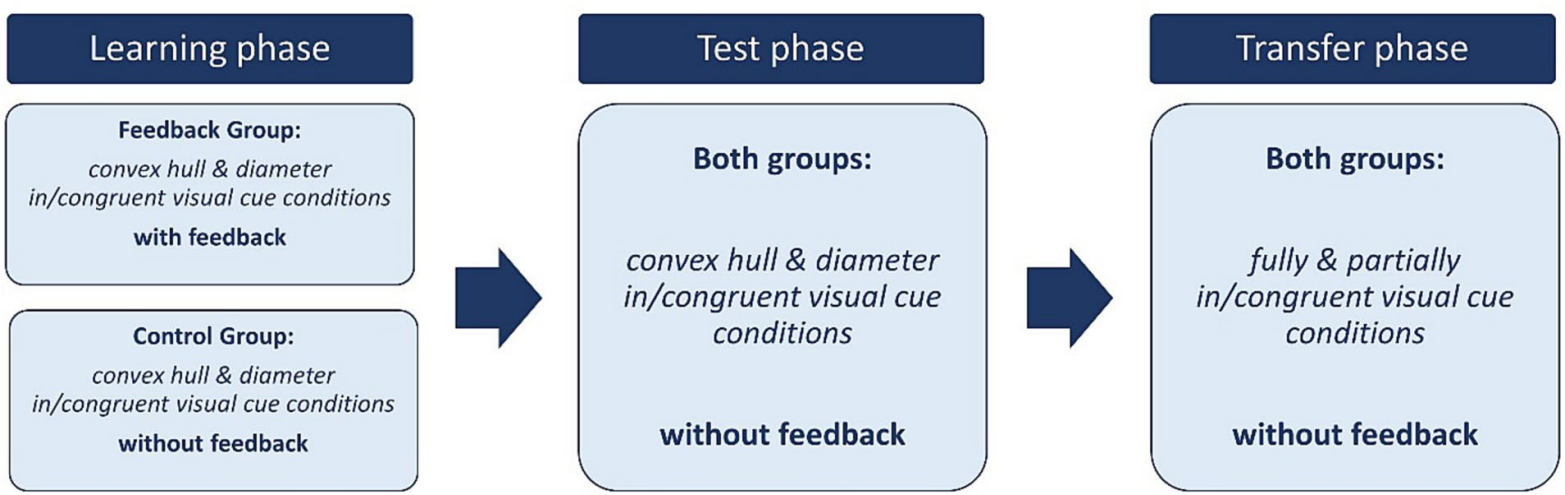
Experimental design of the dot comparison task. This figure shows the experimental design of the dot comparison task. In the learning phase the visual cue conditions *convex hull in/congruent* and *diameter in/congruent* were presented. During this phase the experimental group received feedback after each trial indicating whether their response was correct or incorrect while the control group did not receive any feedback. In the following test phase, the *convex hull in/congruent* and *diameter in/congruent* visual cue conditions were presented again but neither group received feedback. In the transfer phase the *fully in/congruent* and *partially in/congruent* visual cue conditions were presented without feedback in both groups.

The task was divided into three phases: *learning phase, test phase,* and *transfer phase* as shown in Figure 3. In each phase, the different visual cue conditions were presented in a mixed fashion, i.e., the conditions used alternated randomly within each phase. The mixed presentation was chosen because Pekár and Kinder’s (2020) study suggests that presenting visual cue conditions this way increases congruency effects. Since our study aims to examine changes in overall performance as well as changes in congruency effects, increased congruency effects may be beneficial to make changes more visible. A total of 192 trials (96 congruent and 96 incongruent trials) were created for each visual cue condition. To avoid showing the same images for the learning and test phases, two parallel versions were created by dividing the *convex hull in/congruent* and *diameter in/congruent* conditions in half. Thus, the learning and test phases each consisted of 96 stimuli of the *convex hull in/congruent* condition and 96 stimuli of the *diameter in/congruent* condition. The parallel versions used in the learning phase and in the test phase were counterbalanced across participants. The procedure of the experimental and control groups differed only during the learning phase. The experimental group received feedback after each trial during the learning phase whereas the control group did not. Participants in the experimental group were shown the German words “Richtig!” or “Falsch!” (meaning correct or incorrect in German, respectively) for a duration of 500 ms after responding while the control group was shown a blank screen for 500 ms. During the test phase and the transfer phase neither group received feedback and there were no further differences between them in terms of tasks.

**Figure 4:**
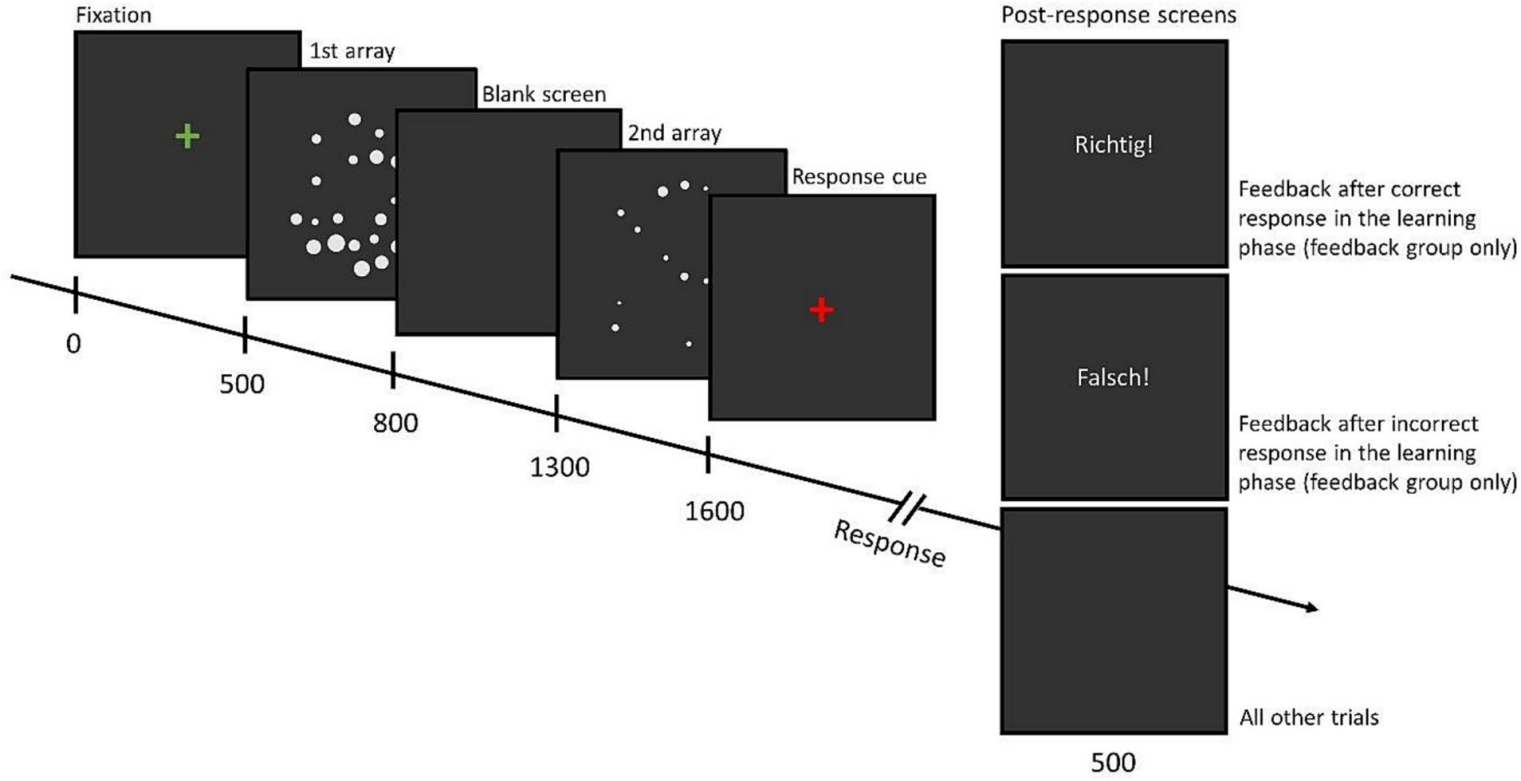
Example of trials in the dot comparison task with timing information. The figure shows an example of trials in the dot comparison task. During the learning phase, participants in the experimental group received feedback (“Richtig!” or “Falsch!,” meaning correct or incorrect in German, respectively) for a duration of 500 ms after responding while the control group did not receive any feedback. This group was shown a blank screen for 500 ms. For the remainder of the task (test phase and transfer phase) both groups were presented with a blank screen for 500 ms after pressing a response button. Times are given in milliseconds.

The procedure of a single trial is shown in Figure 4. First, a green fixation cross was presented for 500 ms, then the first dot array was presented for 300 ms, a blank screen for 500 ms, followed by the second array of dots that was also displayed for 300 ms. Next, a red fixation cross was presented until participants responded. There was no time limit for the participants to respond. They were instructed to press the left control key on the keyboard if the first image contained more dots and the right control key if the second image contained more dots. After the response, depending on the phase and group, either feedback was given or a blank screen was displayed for 500 ms. Accuracy data were obtained and analyzed. During the experiment, the presentation of the trials was divided into blocks of 96 trials. Completing one block took approximately 5 minutes. In total 768 image pairs were shown to the participants (192 for each of the four visual manipulation conditions). The learning phase and the test phase each contained 192 trials (96 trials for the *convex hull in/congruent* and 96 trials for the *diameter in/congruent* conditions, presented in separate blocks). The transfer phase contained 384 trials divided into blocks of 96 trials (192 trials for the *fully in/congruent* and 192 trials for the *partially in/congruent* conditions, presented intermixed).

Upon receiving the instructions, participants completed a practice block with 6 trials. The goal of the practice block was to ensure that they understood the task and the key-to-response correspondence. Thus, participants were provided with feedback regarding whether their response was correct or incorrect and the numerosities were chosen to be very easy to discriminate (8 and 48). All participants received the same practice trials with feedback, irrespective of whether they had been assigned to the control group or to the experimental group. After the practice block, participants were administered the task as described above and were informed about the absence of feedback when necessary. They could take a break of any length at the end of each block. In total, the dot comparison task took approximately 50 minutes.

### 2.5 Arithmetic task

A computer-based mental arithmetic task was used to measure mathematical ability. The task was a replication of the mental arithmetic task used in a study by Lyons and Beilock (2011). The test was divided into four blocks that were presented in the same order to all participants (1) addition, (2) subtraction, (3) multiplication, and (4) division. Participants typed their answer on the number pad of the keyboard. They could correct their answer with the backspace key and confirmed their response with the return key. The problems had to be solved mentally without making notes. In the addition block the arithmetic problems consisted of three one- or two-digit addends and two- or three-digit results. In the subtraction block subjects had to solve subtraction problems in which two two-digit numbers were presented and a two-digit difference had to be calculated. In the multiplication block two two- or three-digit numbers were presented of which the product was always a two- or three-digit number. In the division block the dividend was always a two- or three-digit number, the divisor was always a one-digit number. The results were always two- or three-digit numbers.

Each block lasted 3 minutes and the number of problems participants solved correctly within this 3-minute time window was counted. Each block started with 5 practice trials with feedback. Math problems were always presented in the same order for all participants, and they increased in difficulty. To obtain an arithmetic score for each participant, the number of correct answers for each of the four blocks was z-transformed and the mean of these four values was calculated. This arithmetic score was then used in further analyses. Completing the arithmetic task took approximately 15 minutes.

### 2.6 Statistical analyses

All statistical analyses were performed using R Studio 1.1.456 (R Core Team, 2019). For the dot comparison task accuracy was calculated as the percentage of correct responses for each participant, group (control vs. experimental), visual cue condition (*convex hull*, *diameter*, *fully*, and *partially in/congruent*), congruency (congruent vs. incongruent) and phase (learning, test, and transfer).

To determine whether accuracy differed between the experimental and control groups, a 2 × 3 mixed-design analysis of variance (ANOVA) was conducted with group as between-subject factor (control vs. experimental) and phase as within-subject factor (learning vs. test vs. transfer). To analyze congruency effects in the three phases and to determine whether they were affected by feedback, three 2 × 2 × 2 ANOVAs were conducted separately for the learning, test, and transfer phases with group as between-subject factor (control vs. experimental) and the within-subject factors visual cue condition (for learning phase and test phase: *convex hull in/congruent* and *diameter in/congruent*, for transfer: *fully in/congruent* and *partially in/congruent*) and congruency (congruent vs. incongruent). Follow-up analyses were performed for significant main effects and interaction effects.

The inhibition and the arithmetic score were correlated with overall performance on the dot comparison task averaged across the test and transfer phases. Additionally, inhibition and arithmetic scores were correlated with accuracy in congruent and incongruent trials from the *convex hull* and *diameter in/congruent* visual cue conditions separately. *Fully* and *partially in/congruent* trials were not examined separately because both visual cues are manipulated simultaneously, so it is not entirely clear how to distinguish between congruent and incongruent trials.

## 3 Results

### 3.1 Dot comparison task

#### 3.1.1 Overall performance

**Figure 5:**
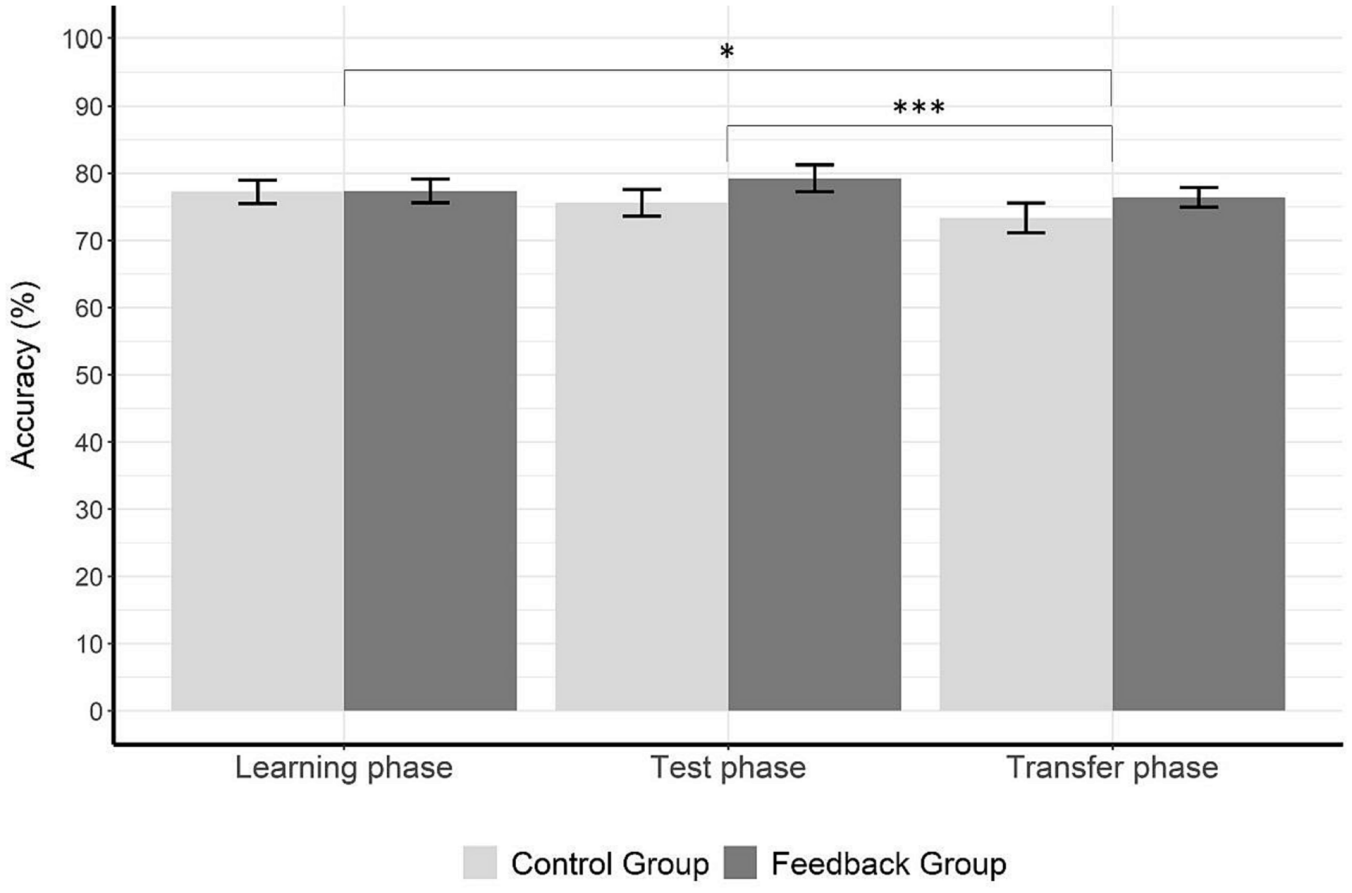
Accuracy data for each phase and group. The figure shows accuracy (%) for each phase and group. There is a significant difference between the learning phase and transfer phase, as well as between test phase and the transfer phase. The control group (no feedback given in the learning phase) is displayed in light gray and the experimental group (feedback given in the learning phase) is displayed in dark gray. Error bars represent the standard error of means. Asterisks indicate significance levels with *p* < 0.05*, *p* < 0.01**, and *p* < 0.001***.

Figure 5 shows percentages of correct responses for both groups in all three phases of the dot comparison task. The 2 × 3 ANOVA with the factors group (control vs. experimental) and phase (learning vs. test vs. transfer) revealed a significant main effect of phase, *F*(2,76) = 4.96, *p* = 0.009, $\eta_{p}^{2}$ = 0.12, but no significant main effect of group, *F*(1,38) = 0.87, *p* = 0.36, $\eta_{p}^{2}$ = 0.02. To follow up the main effect of phase, we calculated three pairwise *t*-tests and Bonferroni-Holm adjusted the significance levels between the accuracies of each phase. These post-hoc comparisons revealed a significant difference in accuracy between the learning phase (*M* = 77.30, *SD* = 7.82) and the transfer phase (*M* = 74.88, *SD* = 8.43), *t*(39) = 2.38, *p* = 0.022, *d* = 0.38, as well as between the test phase (*M* = 77.42, *SD* = 8.95) and the transfer phase, *t*(39) = 3.56, *p* < 0.001, *d* = 0.56. As shown in Figure 5, participants in both groups responded significantly less accurately during the transfer phase than during the learning phase and test phase.

#### 3.1.2 Learning phase

**Figure 6:**
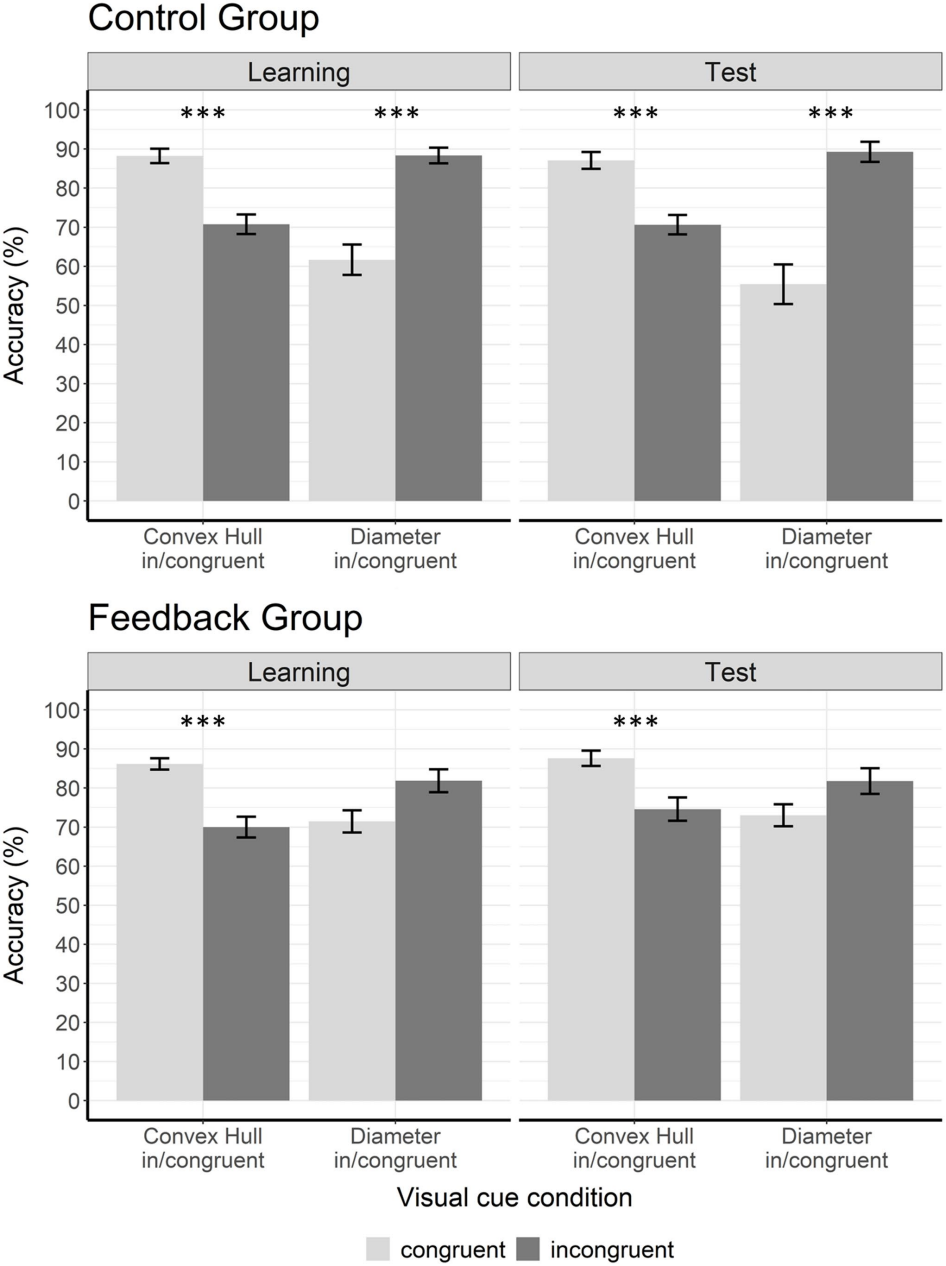
Accuracy data for learning phase and test phase. The figure shows accuracy (%) in the learning phase and in the test phase for both groups. In the control group, the congruency effects were significant in the *convex hull in/congruent* as well as in the *diameter in/congruent* visual cue conditions during learning phase and test phase. In the experimental group, the *convex hull in/congruent* congruency effect was significant during learning phase and test phase but the *diameter in/congruent* congruency effect was significant only during learning phase. Congruent trials are displayed in light gray and incongruent trials in dark gray. Error bars represent the standard error of mean. Asterisks indicate significant results with *p* < 0.05*, *p* < 0.01**, and *p* < 0.001***.

Figure 6 (left panels) shows the accuracy data of both groups in the learning phase separately for congruent and incongruent trials. The 2 × 2 × 2 ANOVA with the factors group (control vs. experimental), visual cue condition (*convex hull in/congruent* vs. *diameter in/congruent*), and congruency (congruent vs. incongruent) revealed a main effect of visual cue condition, *F*(1,38) = 8.27, *p* = 0.007, $\eta_{p}^{2}$ = 0.18 and a two-way interaction between group and congruency, *F*(1,38) = 5.65, *p* = 0.023, $\eta_{p}^{2}$ = 0.13. Congruency effects were different for the two visual cue conditions, as indicated by the two-way interaction between visual cue and congruency, *F*(1,38) = 81.52, *p* < 0.001, $\eta_{p}^{2}$ = 0.68. The difference in congruency effects between the two visual cue conditions was modulated by group, as indicated by a significant three-way interaction between group, visual cue condition and congruency, *F*(1,38) = 5.05, *p* = 0.031, $\eta_{p}^{2}$ = 0.12.

To follow up the three-way interaction, we conducted two 2 × 2 ANOVAs with factors group (control vs. experimental) and congruency (congruent vs. incongruent) separately for *convex hull in/congruent* and *diameter in/congruent* visual cue conditions. For the *convex hull in/congruent* condition, the analysis revealed a significant main effect of congruency, *F*(1,38) = 94.00, *p* < 0.001, $\eta_{p}^{2}$ = 0.71. This main effect was due to a better performance on *convex hull congruent* trials (*M* = 87.19, *SD* = 7.41) than on *convex hull incongruent* trials (*M* = 70.36, *SD* = 11.38). The congruency effects did not differ between the groups, as indicated by a non-significant congruency x group interaction, *F*(1,38) = 0.15, *p* = 0.699, $\eta_{p}^{2}$ = 0.004.

For the *diameter in/congruent* condition, the 2 × 2 ANOVA revealed a significant main effect of congruency, *F*(1,38) = 35.93, *p* < 0.001, $\eta_{p}^{2}$ = 0.49, and a significant interaction of group and congruency, *F*(1,38) = 6.90, *p* = 0.012, $\eta_{p}^{2}$ = 0.15. This interaction was due to a significantly larger congruency effect in the control group (congruent: *M* = 61.67, *SD* = 17.37, incongruent: *M* = 88.33, *SD* = 8.87) than in the feedback group (congruent: *M* = 71.46, *SD* = 12.65, incongruent: *M* = 81.88, *SD* = 13.13). We calculated four post-hoc *t*-tests (with Bonferroni-Holm adjusted *α* levels). First, we calculated two pairwise *t*-tests to see if congruency effects were significant in both groups. The analyses showed a significant congruency effect in the control group, *t*(19) = −5.77, *p* < 0.001, *d* = −1.29 (Bonferroni-Holm adjusted *α level* = 0.0125), but no significant congruency effect in the feedback group, *t*(19) = −2.54, *p* = 0.020, *d* = −0.57 (Bonferroni-Holm adjusted *α* level = 0.016). This significant congruency effect in the control group was due to better performance on incongruent trials than on congruent trials (Figure 5). Second, to determine whether the difference in congruency effects between groups was due to congruent or incongruent trials, we conducted two *t*-tests for independent groups. The assumption of equal variances was not violated as indicated by Levene’s test. There were no significant differences between congruent trials in the control group and congruent trials in the feedback group, *t*(38) = −2.04, *p* = 0.049, *d* = 0.64 (Bonferroni-Holm adjusted α level = 0.025) and no significant differences between incongruent trials in the control group and incongruent trials in the feedback group, *t*(38) = 1.82, *p* = 0.076, *d* = −0.58 (Bonferroni-Holm adjusted α level = 0.05). In sum, in the learning phase the congruency effect in the *diameter in/congruent* condition was significant in the control group. In the experimental group it was significantly smaller to the point of not reaching significance anymore.

#### 3.1.3 Test phase

Figure 6 (right panels) shows accuracy data during the test phase separately for congruent and incongruent trials. Note that no feedback was given in the test phase. Thus, differences between the groups were due to learning processes that occurred in the learning phase. We performed a 2 × 2 × 2 ANOVA with the between-subject factor group (control vs. experimental) and the within-subject factors visual cue condition (*convex hull in/congruent* vs. *diameter in/congruent*) and congruency (congruent vs. incongruent). Results revealed a main effect of visual cue condition, *F*(1,38) = 16.02, *p* < 0.001, $\eta_{p}^{2}$ = 0.03, and a two-way interaction between visual cue condition and congruency, *F*(1,38) = 65.59, *p* < 0.001, $\eta_{p}^{2}$ = 0.63. There was also a two-way interaction between group and congruency, *F*(1,38) = 6.96, *p* = 0.012, $\eta_{p}^{2}$ = 0.16, indicating differences in congruency effects between groups. These differences depended on the visual cue condition, as indicated by the three-way interaction between group, visual cue condition, and congruency, *F*(1,38) = 10.28, *p* = 0.003, $\eta_{p}^{2}$ = 0.21. The two-way interaction of visual cue and group did not reach significance, *F*(1,38) = 1.22, *p* = 0.28, $\eta_{p}^{2}$ = 0.03.

To follow up on the three-way interaction we conducted two 2 × 2 ANOVAS with the factors group and congruency separately for each visual cue condition (*convex hull in/congruent* and *diameter in/congruent*). For the *convex hull in/congruent* condition, the 2 × 2 ANOVA (control vs. experimental × congruent vs. incongruent) revealed a significant main effect of congruency, *F*(1,38) = 64.72, *p* < 0.001, $\eta_{p}^{2}$ = 0.63. This main effect of congruency was due to better performance on congruent trials (*M* = 87.34, *SD* = 9.13) than on incongruent trials (*M* = 72.60, *SD* = 12.28) in both groups as can be seen in Figure 6. The group × congruency interaction did not reach significance *F*(1,38) = 0.88, *p* = 0.35, $\eta_{p}^{2}$ = 0.02.

For the *diameter in/congruent* condition, the 2 × 2 ANOVA (control vs. experimental × congruent vs. incongruent) revealed a significant main effect of congruency, *F*(1,38) = 30.30, *p* < 0.001, $\eta_{p}^{2}$ = 0.44, and a significant interaction between group and congruency, *F*(1,38) = 10.52, *p* = 0.002, $\eta_{p}^{2}$ = 0.22. Thus, congruency effects differed between groups. The congruency effect was significantly larger in the control group (congruent: *M* = 55.42, *SD* = 22.57, incongruent: *M* = 89.27, SD = 11.54) than in the feedback group (congruent: *M* = 73.02, *SD* = 12.62, incongruent: *M* = 81.77, *SD* = 14.79).

To further investigate the group × congruency interaction in the *diameter in/congruent* condition, we conducted four *t*-tests using the Bonferroni-Holm correction method. Adjusted α levels are displayed in Table 2. Two pairwise *t*-tests were conducted to compare accuracies on congruent and incongruent trials, separately for each group. The results showed that the congruency effect was only significant in the control group *t*(19) = −5.4, *p* < 0.001, *d* = −1.21. This was because of a significantly better performance on incongruent than on congruent trials in this group. In contrast, the congruency effect in the feedback group was not significant *t*(19) = −1.93, *p* = 0.07, *d* = −0.43. To determine whether the difference in congruency effects between groups was due to congruent or incongruent trials, we conducted two *t*-tests for independent groups. When the assumption of equal variances was violated (as indicated by Levene’s test), we used the Welch test, and adjusted degrees of freedom accordingly. The performance on *diameter congruent* trials differed significantly between groups, *t*(29.82) = 3.04, *p* = 0.005, *d* = 0.96. Participants who had received feedback during training performed significantly better on congruent trials (*M* = 73.0; SD = 12.6) presented during the test phase than participants who had not received feedback (*M* = 55.4, SD = 22.6). There was no significant difference between groups on *diameter incongruent* trials *t*(38) = −1.79, *p* = 0.08, *d* = −0.57. In summary, whether a congruency effect was observed in the *diameter in/congruent* condition during test phase depended on whether participants had received feedback during learning or not. The effect of feedback was only observed on *diameter congruent* trials, i.e., increased accuracy, but not on *diameter incongruent* trials.

**Table 2:** Post-hoc *t*-test in the dot comparison task.

Test: *Diameter in/congruent*	*t* value	*p* value	*α* level
Control congruent vs. incongruent	*t*(19) = −5.4	0.00003***	0.0125
Feedback congruent vs. incongruent	*t*(19) = −1.93	0.07	0.025
Control congruent vs. Feedback congruent	*t*(29.82) = −3.04	0.005**	0.0167
Control incongruent vs. Feedback incongruent	*t*(38) = 1.79	0.08	0.05

Transfer: *Fully in/congruent*	*t* value	*p* value	*α* level
Control: congruent vs. incongruent	*t*(19) = −1.37	0.19	0.05
Feedback: congruent vs. incongruent	*t*(19) = 3.75	0.001**	0.0125
Control congruent vs. Feedback congruent	*t*(27.59) = −2.85	0.008**	0.025
Control incongruent vs. Feedback incongruent	*t*(38) = 2.83	0.007**	0.0167

Transfer: *Partially in/congruent*	*t* value	*p* value	*α* level
Control: congruent vs. incongruent	*t*(19) = −8.36	0.00000008***	0.0125
Feedback: congruent vs. incongruent	*t*(19) = −4.84	0.0001***	0.0167
Control congruent vs. Feedback congruent	*t*(38) = −2.52	0.016*	0.025
Control incongruent vs. Feedback incongruent	*t*(38) = 1.44	0.16	0.05

This table shows the results of the post-hoc *t*-tests on the test phase and transfer phase data. For α levels Bonferroni-Holm corrections were used and *p* values were sorted accordingly. In the *t*-tests for independent groups, degrees of freedom were adjusted using the Welch test when the assumption of equal variances was violated. Significant *p* values are displayed in bold font. Asterisks indicate significant results with *p* < 0.05*, *p* < 0.01**, and *p* < 0.001***.

#### 3.1.4 Transfer phase

**Figure 7:**
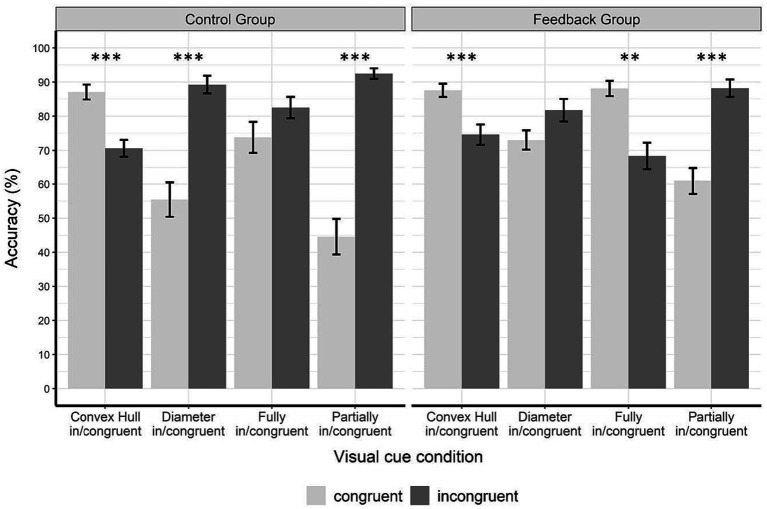
Accuracy data for every visual cue conditions in the test phase and in the transfer phase for each group and congruency condition. The figure shows accuracy (%) in the test phase (*convex hull in/congruent* and *diameter in/congruent* conditions) and in the transfer phase (*fully in/congruent* and *partially in/congruent* conditions). Congruent trials are displayed in light gray and incongruent ones in dark gray. In the control group, there were significant congruency effects for *convex hull in/congruent* trials, *diameter in/congruent* trials, and *partially in/congruent* trial. In the feedback group, there were significant congruency effects for *convex hull in/congruent* trials, *fully in/congruent* trials, and *partially in/congruent* trials. Note that the pattern in the control group equals the pattern in Figure 2A. However, the pattern in the feedback group equals the pattern in Figure 2C. Error bars represent the standard error of mean. Asterisks indicate significant results with *p* < 0.05*, *p* < 0.01**, and *p* < 0.001***.

Figure 7 displays the accuracies of the control group (left panel) and the feedback group (right panel) during the transfer phase (*fully in/congruent* and *partially in/congruent* visual cue conditions). Note that no feedback was given in the transfer phase. Figure 7 additionally shows the data from the test phase (*convex hull in/congruent* and *diameter in/congruent* visual cue conditions) which also can be seen in Figure 6. The combination of the two phases in one figure provides the complete set of visual cue conditions used in the original study by Gebuis and Reynvoet (2012a see also Pekár and Kinder, 2020) and allows to compare the pattern of results with Figure 2.

We conducted a 2 × 2 × 2 ANOVA with group as between-subject factor (control vs. feedback) and the within-subject factors visual cue condition (*fully in/congruent* vs. *partially in/congruent*) and congruency (congruent vs. incongruent). The results revealed a main effect of visual cue condition, *F*(1,38) = 26.56, *p* < 0.001, $\eta_{p}^{2}$ = 0.41, and a main effect of congruency, *F*(1,38) = 19.00, *p* < 0.001, $\eta_{p}^{2}$ = 0.33. We also found a two-way interaction of group and visual cue condition, *F*(1,38) = 5.43, *p* = 0.025, $\eta_{p}^{2}$ = 0.13, a two-way interaction of group and congruency, *F*(1,38) = 11.16, *p* < 0.002, $\eta_{p}^{2}$ = 0.23, and a two-way interaction of visual cue condition and congruency, *F*(1,38) = 149.10, *p* < 0.001, $\eta_{p}^{2}$ = 0.78. The two-way interaction of group and congruency indicates that congruency effects depended on whether participants had received feedback in the learning phase. Furthermore, the two-way interaction between visual cue condition and congruency reveals that congruency effects differed between the two visual cue conditions. The three-way interaction of visual cue congruency and group was not significant *F*(1,38) = 1.24, *p* = 0.27, $\eta_{p}^{2}$ = 0.03.

Because of the two interactions that included visual cue condition, we calculated two separate 2 × 2 ANOVAS for the *fully* and *partially in/congruent* conditions with group (control vs. feedback) and congruency (congruent vs. incongruent) as between-and within-subject factors, respectively. In the *fully in/congruent* condition there was a significant interaction between group and congruency, *F*(1,38) = 11.84, *p* = 0.001, $\eta_{p}^{2}$ = 0.24, the congruency main effect *F*(1,38) = 1.77, *p* = 0.19, $\eta_{p}^{2}$ = 0.05 and the group main effect *F*(1,38) = 0.00, *p* = 0.985, $\eta_{p}^{2}$ = 0.00 did not reach significance. The significant group × congruency interaction indicates that congruency effects in the *fully in/congruent* condition differed significantly between groups. As can be seen in Figure 7, this effect is due to a difference in the direction of the congruency effect between groups. Descriptively, the mean accuracy in the control group was higher on incongruent trials than on congruent ones (congruent: *M* = 73.80, *SD* = 20.2, incongruent: *M* = 82.55, *SD* = 14.00), and the opposite pattern was observed for the feedback group. In this group, descriptively, the mean accuracy was higher on congruent trials than on incongruent ones (congruent: *M* = 88.13, *SD* = 9.90, incongruent: *M* = 68.33, *SD* = 17.50). In order to test for significance of these differences and to further investigate the group × congruency interaction in the *fully in/congruent* condition, we performed four *t*-tests using the Bonferroni-Holm correction method. Adjusted α levels are displayed in Table 2. Two pairwise *t*-tests were conducted to compare accuracies on congruent and incongruent trials separately for each group. The congruency effect in the control group, in which performance was better on incongruent than on congruent trials, was not significant, *t*(19) = −1.37, *p* = 0.19, *d* = −0.31. This finding is in line with previous studies by Gebuis and Reynvoet (2012a) and Pekár and Kinder (2020). The lack of a congruency effect in this condition can be explained by the opposite effects of diameter and convex hull, which cancel each other out, as both visual cues are manipulated in the same direction. This congruency effect, however, was significant in the feedback group, *t*(19) = 3.75, *p* = 0.001, *d* = 0.84, indicating that in this group performance was better on congruent than on incongruent trials. Thus, in the feedback group, the direction of the congruency effect was the same in the *fully in/congruent* condition as in the *convex hull in/congruent* condition. To examine whether differences in congruency effects were caused by congruent or incongruent trials, we used two *t*-tests for independent groups. When the assumption of equal variances was violated (as shown by Levene’s test), we used the Welch test, and adjusted degrees of freedom accordingly. On *fully congruent* trials, participants in the feedback group performed significantly better than participants in the control group, *t*(27.59) = −2.85, *p* = 0.008, *d* = −0.90. On *fully incongruent* trials, however, participants in the control group performed significantly better than participants in the feedback group, *t*(38) = 2.83, *p* = 0.007, *d* = 0.88. Thus, both congruent and incongruent trials contributed to the finding that there was a significant congruency effect in the feedback group, but no effect in the control group.

The 2 × 2 ANOVA for the *partially in/congruent* condition (control vs. experimental × congruent vs. incongruent) revealed a significant main effect of congruency, *F*(1,38) = 87.57, *p* < 0.001, $\eta_{p}^{2}$ = 0.70, and a significant interaction between congruency and group, *F*(1,38) = 6.61, *p* = 0.014, $\eta_{p}^{2}$ = 0.15. As both congruency effects were in the same direction, this effect indicates that the congruency effect was significantly larger in the control group (congruent: *M* = 44.53, *SD* = 23.3, incongruent: *M* = 92.50, *SD* = 6.90) than in the feedback group (congruent: *M* = 60.94, *SD* = 17.3, incongruent: *M* = 88.23, *SD* = 11.3).

To further investigate the group × congruency interaction in the *partially in/congruent* condition, we performed four *t*-tests using the Bonferroni-Holm correction method. Adjusted α levels are displayed in Table 2. There was a significant congruency effect in the control group, *t*(19) = −8.36, *p* < 0.001, *d* = −1.87. This effect was due to better performance on incongruent (*M* = 44.5, *SD* = 23.3) than on congruent trials (*M* = 92.5, *SD* = 6.9). This finding is in line with previous studies by Gebuis and Reynvoet (2012a) and Pekár and Kinder (2020). It can be explained by the opposite effects of convex hull and diameter that are cumulated as the two visual cues are manipulated in opposite directions in this condition. In the feedback group this congruency effect of participants performing significantly better on incongruent than on congruent trials was also found, *t*(19) = −4.84, *p* < 0.001, *d* = −1.08, although it was significantly smaller than in the control group (as shown by the ANOVA results above). To examine if the difference in the congruency effects was caused by congruent or incongruent trials, we conducted two *t*-tests for independent groups. When the assumption of equal variances was violated (as shown by Levene’s test), we used the Welch test, and adjusted degrees of freedom accordingly. There was a significant difference between groups for *partially congruent* trials (diameter congruent, convex hull incongruent), *t*(38) = −2.52, *p* = 0.016, *d* = −0.80. The feedback group performed significantly better in *partially congruent* trials than the control group. There was no significant difference in performance in *partially incongruent* trials (diameter incongruent, convex hull congruent) between groups *t*(38) = −1.44, *p* = 0.016, *d* = −0.46.

### 3.2 Correlations

The arithmetic score correlated significantly only with accuracy on *diameter incongruent* trials, *t*(37) = 2.88, *r* = 0.43, *p* = 0.007 (see Table 3). No significant difference was observed in that correlation between groups, *z* = 0.83, *p* = 0.406. We found no significant correlation between the inhibition score obtained from the Color Stroop task and any variable from the dot comparison task (Table 3).

**Table 3:** Pearson correlation coefficients of the variables from the dot comparison task with arithmetic and inhibition scores.

Dot Comparison variable	Arithmetic score	Inhibition score
Overall performance	0.29 (0.08)	−0.05 (0.74)
Convex hull congruent	0.27 (0.09)	−0.18 (0.28)
Convex hull incongruent	0.29 (0.07)	0.06 (0.74)
Diameter congruent	0.02 (0.88)	−0.02 (0.89)
Diameter incongruent	0.43 (0.01)**	−0.01 (0.59)

Significant coefficients are displayed in bold font. Asterisks represent significance levels with *p* < 0.05*, *p* < 0.01**, and *p* < 0.001***.

## 4 Discussion

The aim of the present study was to investigate the role of learning in non-symbolic numerical processing. Specifically, we were interested in the influence of continuous visual properties on numerical comparisons and how this influence is affected by learning. To this end, we introduced feedback into the classical dot comparison task and examined how feedback-induced learning affected performance in different visual cue manipulation conditions. We also examined how changes in performance were related to inhibitory and mathematical ability.

First, overall performance was not affected by the presence or absence of feedback in the learning phase, as the accuracy levels of the experimental and the control groups were similar in all three phases. The only difference found in overall performance was a decrease in accuracy in the transfer phase which can be attributed to the more complex nature of the trials in the *fully in/congruent* and *partially in/congruent* conditions. In both conditions, two visual properties – convex hull and diameter – were manipulated simultaneously.

Second, we examined how feedback affected congruency effects in the test and transfer phases by comparing performance on congruent and incongruent trials in the different visual cue conditions. In the control group, we were able to replicate the pattern of congruency effects previously reported in this dot comparison task (Gebuis and Reynvoet, 2012a; Pekár and Kinder, 2020). Specifically, we found a positive congruency effect in the *convex hull in/congruent* condition, a reversed congruency effect in the *diameter in/congruent* condition, no congruency effect in the *fully in/congruent* condition, and an enhanced congruency effect in the *partially in/congruent* condition. However, a different pattern of congruency effects emerged in the participants that had received feedback during the learning phase. Analysis of their performance in the test phase showed that convex hull was not affected by feedback, i.e., the same pattern of congruency effect was found in both groups. However, when looking at the *diameter in/congruent* condition, the congruency effect in the experimental group was not only significantly smaller than in the control group, but also failed to reach significance. While participants in the control group performed significantly better on incongruent than on congruent trials, this reversed diameter effect disappeared completely in the feedback group, as there was no significant difference between congruent and incongruent trials in this group. This disappearance of the congruency effect in the feedback group can be attributed to better performance on congruent trials in this group compared to the control group, while the performance on incongruent trials did not differ between the groups. The analysis of performance in the transfer phase – when the two visual cues were manipulated simultaneously – showed that in the *fully in/congruent* condition the congruency effect changed its direction and became significant: participants in the feedback group performed significantly better on congruent than on incongruent trials. The congruency effect in the control group remained non-significant. Finally, in the *partially in/congruent* condition, the direction of the congruency effect was the same in the two groups. Participants in both groups performed better on *partially incongruent* trials (incongruent on diameter, congruent on convex hull) than on *partially congruent* trials (congruent on diameter and incongruent on convex hull). However, this effect was significantly reduced in the feedback group compared to the control group which was due to a significant difference between feedback and control group on *partially congruent* trials. Participants in the feedback group performed better on these trials than participants in the control group.

Taken together, this pattern of change in congruency effects as a result of feedback delivers important implications about the influence of visual cues and learning in numerosity comparison. In particular, the disappearance of the reversed congruency effect in the *diameter in/congruent* condition as a result of the provided feedback is an important finding. Note, that none of the visual cues were informative of numerosity either in any of the visual cue conditions or throughout the entire experiment. Thus, the diminished congruency effect of diameter means that participants in this group learned from the feedback that diameter was not informative about numerosity in this task. Whereas the preserved congruency effect of convex hull, on the other hand, shows that the same knowledge was not acquired about convex hull. Despite the feedback, participants continued to rely on this cue in the same way as those in the control group. These results provide evidence that the influence of a certain visual cue – in this case dot diameter – on non-symbolic numerosity processing is not fixed but subject to change under specific conditions based on prior experience. However, it seems that the influence of convex hull is more robust and less susceptible to change under the same conditions.

Furthermore, participants who had received feedback during the learning phase appeared to have applied their knowledge, that diameter was not informative of numerosity, to the transfer phase. In this phase, diameter and convex hull were manipulated together within each trial in the *fully in/congruent* and in the *partially in/congruent* conditions. According to Gebuis and Reynvoet (2012a) the originally observed congruency effects in these conditions – which we were able to replicate in the control group – are due to the additive integration of the two individual congruency effects. In particular, they suggest that when convex hull and diameter are manipulated simultaneously, the congruency effect in the *convex hull in/congruent* and the reversed congruency effect in the *diameter in/congruent* condition are added together. This results in an enhanced congruency effect in the *partially in/congruent* condition – when convex hull and diameter are manipulated in opposite directions – and in the absence of the congruency effect in the *fully in/congruent* condition when convex hull and diameter are manipulated together in the same direction. Remarkably, the same additive process was observed in the feedback group. However, in this case the combination of the preserved congruency effect of convex hull and the diminished congruency effect of diameter were added up when the two visual cues were manipulated together. More specifically, in the *fully in/congruent* condition the direction of the congruency effect changed and reached significance in the feedback group. Participants performed significantly better on congruent trials than on incongruent trials. Not only the direction but also the approximate size of this congruency effect corresponds to the direction and the size of the congruency effect observed in the *convex hull in/congruent* condition. Again, this change in the congruency effect further confirms the idea that participants in the experimental group learned that diameter is not informative of numerosity. As a result of the additive weighing process as proposed by Gebuis and Reynvoet (2012a), the preserved congruency effect of convex hull and the diminished congruency effect of diameter were added together in the experimental group resulting in a congruency effect equal to the individual congruency effect of convex hull (see Figure 2C).

In the *partially in/congruent* condition the congruency effect was significantly smaller in the feedback group than in the control group. Once more, this result can be explained with the additive weighing process put forward by Gebuis and Reynvoet (2012a) but in this case the preserved congruency effect of convex hull and the diminished congruency effect of diameter were added together when they were manipulated in the opposite direction. Since the reversed congruency effect of diameter disappeared in the feedback group, adding it together with the *preserved* congruency effect of convex hull produced a smaller congruency effect than in the control group. Taken together, this pattern of results in the experimental group clearly indicates that participants learned from feedback that diameter was not informative of numerosity in this task. Moreover, this knowledge was then transferred to the remainder of the task as manipulating this cue did not affect any of the congruency effects in the different visual cue conditions.

In our study, however, learning was limited to the average diameter of the dots, while the convex hull of the dot patterns continued to influence numerosity comparison. This finding suggests that the weight assigned to the convex hull of dot patterns remained the same even though participants could learn that it was not informative about numerosity. In contrast, the weight assigned to the average diameter of the dots appeared to be significantly reduced by learning. This interpretation is consistent with a proposal by Pekár and Kinder (2020) that diameter and convex hull are processed in inherently different ways when participants make numerosity judgments. Since there are no neurobiological correlates of the reversed congruency effect of diameter, Pekár and Kinder (2020) argued that this effect is unlikely to be innate, but rather may be the result of learning. Such a learning process could be based on previous experience that sets of smaller objects tend to be more numerous (Pekár and Kinder, 2020). For example, a bag of a certain size tends to contain more blueberries than apples. Similarly, an aquarium of a certain size will hold more small fish than large fish.

These findings provide strong evidence for the Sensory Integration Theory (Gebuis et al., 2016; Gevers et al., 2016). According to this theory, non-symbolic numerical tasks are solved by assigning weights to visual cues that are integrated through an additive process. The present study extends this theory by showing that the weights assigned to a particular visual cue can be modified by learning. This modification is not a transient phenomenon, present only as long as feedback is given, but has an impact on responses even after feedback has ceased. Most importantly, the altered weights affect responses even when the visual cues in the dot pattern pairs are manipulated in a completely novel way. Even in this case, the patterns of congruency effects can be explained by assuming that weights are combined in an additive manner when making numerosity judgments.

As it has been pointed out, studies investigating the developmental changes in numerical abilities suggest that ANS accuracy improves over time. Such improvements may partially be due to learning about visual cues and adjusting the weights given to them which, in turn, leads to a decrease in their influence and the refinement of the ANS over development. For example, it is conceivable that in the case of the convex hull cue, which has a positive correlation with numerosity in natural scenes (more apples cover more space), the weights do not need to be reassessed through learning because its informational value remains constant. In contrast, in the case of the diameter cue, which often has a negative correlation with numerosity in natural scenes (more blueberries than apples fit in a basket), there may be a greater benefit from learning.

To test this possibility, future studies should investigate whether only diameter or also other visual cues can be influenced by learning. Moreover, it would be useful to further investigate the *convex hull in/congruent* condition to see whether the congruency effect is truly robust or whether it can also be modified and under what circumstances this might happen. In the current study, the *convex hull in/congruent* and *diameter in/congruent* trials were presented in a mixed fashion during the training and test phases, i.e., they alternated randomly. It is possible that this arrangement caused participants to focus on one visual cue, making it more difficult to adjust the weight of the other visual cue. This hypothesis could be tested by slightly modifying the current task and including only *convex hull in/congruent* trials. Although the current study clearly shows that diameter is more susceptible to learning than convex hull, a small change in presentation mode would allow us to investigate whether feedback has any effect on performance and the direction of the congruency effect in the *convex hull in/congruent* condition. If the congruency effect of convex hull is not affected by providing transparent information that convex hull is not informative about numerosity, this may actually be a result of innate properties of the non-symbolic number processing system. The results of such future studies could provide important insights into the additive weighing process proposed by proponents of Sensory Integration Theory, such as whether the weights of all visual cues are modifiable and subjective to prior experience or only the weights of some visual cues.

The cognitive mechanisms underlying the exclusion of the diameter cue from influencing numerosity processing are not yet fully understood. However, viewing our results through the lens of the *sharpening* and *filtering* hypotheses may provide additional insights into the mechanisms involved (Piazza et al., 2018). As mentioned above, the *filtering* hypothesis suggests that non-numerical information is increasingly filtered out during development. Consequently, as numerical processing becomes less influenced by visual properties, performance on incongruent trials, where visual cues interfere, should increase. Performance on congruent trials, where visual cues do not interfere with performance, should remain constant or even decrease. In contrast, the *sharpening* hypothesis suggests that as numerical ability increases during development, number representations become more accurate. As a result, overall error rates should decrease (Piazza et al., 2018). The results of the present study are difficult to reconcile with the *sharpening* hypothesis because performance was equal between groups: Promoting learning through feedback did not improve the overall accuracy of numerosity comparisons. Rather, at first glance, the current results seem to support the *filtering* hypothesis. In the feedback group, congruency effects were significantly smaller in the *diameter in/congruent* condition as well as in the *partially in/congruent* condition, as predicted by the *filtering* hypothesis. However, these reduced congruency effects were caused by an increase in accuracy on *congruent* rather than *incongruent* trials. In fact, accuracy on incongruent trials did not change at all. Thus, while the decrease in the congruency effect is consistent with the *filtering* hypothesis, the direction of this change is not. However, there is a way to reconcile our results with the *filtering* hypothesis. Because of the reversed congruency effect of diameter, congruent trials are actually the more difficult trials, while incongruent trials are the easier trials. This has been shown repeatedly in previous studies (Gebuis and Reynvoet, 2012a; Pekár and Kinder, 2020). In more difficult trials, visual cues are misleading, and must be filtered out, whereas in easier trials they are helpful. Thus, when the congruent trials are the more difficult ones, the *filtering* account would predict that learning improves performance on congruent trials and does not improve performance on incongruent trials. Hence, at a basic conceptual level, our results are consistent with the *filtering* account. However, one problem remains: The *filtering* account considers all visual properties as a single dimension, i.e., it does not distinguish between different visual cues that affect numerosity judgments in different ways. Thus, it does not account for the reversed congruency effect of diameter. As a result, the *filtering* hypothesis in its current form cannot explain the need to filter out the misleading diameter cue on *congruent* trials while there is no such need on *incongruent* trials. However, it would be possible to modify the account to differentiate between different visual cues and their different influences on numerosity judgments.

Regarding the second aim of the current study, we found no evidence that inhibition is related to numerosity processing in dot comparison, as indicated by the lack of correlations between inhibition measured by the Color Stroop task (Bäumler, 1985) and any of the dot comparison variables. These findings are inconsistent with some of the previous studies that suggest a relationship between inhibition abilities and performance in the dot comparison task (Leroux et al., 2006; Houdé et al., 2011; Clayton and Gilmore, 2015). However, they are consistent with a study by Reynvoet et al. (2021) that also failed to find correlations between performance on the dot comparison task using the same type of stimuli as in the present study (Gebuis and Reynvoet, 2012a) and several different inhibitory measures, i.e., number go/nogo task, animal go/nogo task, numerical Stroop task, and animal Stroop task. Reynvoet et al. (2021) argued that the type of dot comparison task could strongly influence the correlation with inhibition tasks. Furthermore, it is important to note that a variety of different inhibition measures have been used in the literature (Clayton and Gilmore, 2015). This makes it even more difficult to compare results across studies. Measuring inhibition is difficult because there are different types of inhibition. An important distinction has been made between interference control such as that measured by the Color Stroop task (Bäumler, 1985) and response inhibition, which can be measured by go/nogo tasks (Nigg, 2000; also Pekár et al., 2023). Furthermore, there may be multiple domain-specific control systems in the human brain (Egner, 2008). This raises the problem of choosing an appropriate measure of inhibition, as it is unclear which type of inhibition is relevant in each dot comparison task. We chose the Color Stroop task to measure inhibition because it does not involve numerosity or other numerosity-related visual parameters. In doing so, we wanted to avoid artificially inflating the correlation that might occur when comparing performance on the dot comparison task with inhibition tasks that involve numerical or magnitude/size information, such as the numerical Stroop task (numerical or physical size of symbolic numbers) or the animal Stroop task (physical size of animals). Based on the current findings, there does not appear to be a relationship between inhibition – in the form of non-numerical interference control – and performance on the specific dot comparison task we used, which was constructed like the one introduced by Gebuis and Reynvoet (2012a).

Regarding our third aim, we found a significant correlation between performance on the mental arithmetic task and performance on *diameter incongruent* trials of the dot comparison task. We found no correlation with any of the other dot comparison measures. In the current literature, results regarding the relationship between arithmetic performance and accuracy on dot comparison tasks are very heterogeneous, with some studies finding a relationship between these two measures and others not (for an overview see Reynvoet et al., 2021). Our results may provide an explanation for such inconsistent findings. Previous studies have used overall performance on the dot comparison task to determine correlations (Sasanguie et al., 2014). However, our study shows that congruency can affect numerical processing in different ways and this effect of congruency depends on the visual cue at hand. Therefore, the use of overall averages does not seem reasonable and it may even be the reason for conflicting results. Furthermore, averaging congruent and incongruent trials separately for each condition is only clear in conditions in which only one visual property is being manipulated like the *convex hull in/congruent* and the *diameter in/congruent* condition. Averaging separately for congruency in *fully* and *partially in/congruent* conditions yields the problem of two visual properties being manipulated simultaneously which have opposite effects on numerosity processing in this task. Hence, identifying which trials are “truly” congruent and incongruent seems arbitrary. Therefore, we argue that to examine the correlation between performance on the dot comparison task and mathematical performance, it is necessary to consider congruent and incongruent trials of the different visual cue conditions separately and to be cautious in making claims about which trials are congruent and incongruent in the *fully* and *partially in/congruent* conditions. But why is performance only on diameter incongruent trials positively correlated with mathematical performance? The correlation found may be related to the learned nature of the reverse congruency effect in the *diameter in/congruent* condition. One possibility is that participants, who learn well from their experience that smaller objects tend to be more numerous in natural scenes, are also better at acquiring mathematical skills. This phenomenon may be governed by domain-general or domain-specific learning processes. However, since our study is the only one so far showing this specific type of positive correlation, it is crucial to replicate the results. It is also crucial to further investigate the exact nature of the relationship between specific types of inhibition and specific types of non-symbolic numerical stimuli. In general, it is important to note that the results regarding the relationship between performance in the dot comparison task and inhibition as well as arithmetic competencies are very heterogeneous in literature and seems to be heavily influenced by the type of dot comparison task used, the measure of inhibition and the measure of arithmetic competencies. Hence, generalizing the findings from this study to other dot comparison, inhibition or arithmetic tasks might be difficult.

In conclusion, this study provides evidence that learning processes can strongly influence numerosity processing in adult participants. We were able to show that the diameter cue can influence non-symbolic numerosity processing to very different extents depending on prior experience. Our results also provide strong evidence for the Sensory Integration Theory of non-symbolic numerical processing: The results are consistent with the notion that weights are assigned to different visual cues and that numerical judgments depend on an additive combination of these weights. Based on our results, the Sensory Integration Theory could be extended to explain the adaptation of these weights as a result of prior experience.

## Data availability statement

The raw data supporting the conclusions of this article will be made available by the authors, without undue reservation.

## Ethics statement

The requirement of ethical approval was waived by Ethics Committee of the Department of Education and Psychology of the Free University Berlin for studies involving humans. The study was conducted in accordance with the local legislation and institutional requirements, as well as with the Declaration of Helsinki. The participants provided written informed consent to participate in this study.

## Author contributions

WH: Conceptualization, Data curation, Formal analysis, Investigation, Methodology, Project administration, Software, Validation, Visualization, Writing – original draft. AK: Conceptualization, Methodology, Resources, Supervision, Validation, Writing – review & editing. JP: Conceptualization, Funding acquisition, Methodology, Software, Supervision, Validation, Visualization, Writing – review & editing.
